# Complete Pathological Response of Human Papillomavirus (HPV)-Related Oropharyngeal Carcinoma After Primary Treatment With Stereotactic Radiotherapy: A Case Report

**DOI:** 10.7759/cureus.92293

**Published:** 2025-09-14

**Authors:** Isaac Kong, Ka-Kit David Yeung, Han Zhang, Justin W Lee

**Affiliations:** 1 Radiation Oncology, McMaster University, Hamilton, CAN; 2 Otolaryngology - Head and Neck Surgery, McMaster University, Hamilton, CAN

**Keywords:** cure, definitive radiation therapy, dose fractionation, head and neck squamous cell cancer, hpv-related oropharyngeal cancer, pathologic complete response, stereotactic ablative radiation, stereotactic ablative radiotherapy fractionation, stereotactic body radiation therapy

## Abstract

Human papillomavirus (HPV)-related oropharyngeal squamous cell carcinomas (OPSCCs) with locally advanced progression are generally treated with curative intent concurrent chemoradiation. This case report of a 51-year-old female highlights the potential for locoregional control with complete pathologic response following primary stereotactic body radiotherapy (SBRT). Previous studies on SBRT for head and neck cancers report variable dose fractionation schemes and inconsistent volume contouring techniques, with most SBRT applications focused on recurrent or post-operative settings rather than definitive primary treatment. In this case, the patient initially presented with palpable left cervical lymphadenopathy. Diagnostic workup confirmed non-keratinizing invasive squamous cell carcinoma of the left base of the tongue, with strong p16 positivity. After declining standard curative intent treatment options, including chemoradiation, the patient later accepted a palliative SBRT regimen of 40 Gy in five fractions weekly, targeting the oropharynx and unilateral neck. The patient ultimately only received 32 Gy in four fractions. Follow-up imaging demonstrated significant tumor regression, but at five months, a palpable L neck mass remained. A subsequent selective neck dissection confirmed complete pathologic response with no residual carcinoma. The patient remained disease-free 68 months after treatment with minimal late toxicities. This case supports the feasibility of using SBRT as a definitive modality for HPV-related OPSCC, highlighting the potential for durable locoregional control with an acceptable toxicity profile. Given the growing interest in both treatment de-escalation for HPV-related OPSCC and the application of SBRT in head and neck cancers, this report suggests a promising, though preliminary, role of SBRT in selected patients.

## Introduction

Locally advanced oropharyngeal squamous cell carcinoma (OPSCC) is usually treated in the primary setting with concurrent chemoradiation using conventional fractionation with daily treatments delivered over seven weeks [1,2]. In certain situations, radiation alone or transoral robotic surgery can also be used for cure [3-5]. Stereotactic body radiotherapy (SBRT) is a technique of delivering radiotherapy wherein high doses of radiation are delivered over a limited number of fractions, typically five or less. High dose gradients are used to create very conformal volumes and spare adjacent tissues [6]. It offers multiple benefits, including higher ablative doses of radiation to the tumor cells, the potential for reduced toxicity, and fewer appointments for patients. In order to safely provide these ablative doses of radiation, there is a need for increased patient immobilization, imaging, and quality assurance. As the technical ability to deliver more precise and higher dose radiotherapy becomes more readily available, there has been an interest in applying SBRT to head and neck cancers.

While not the standard of care, recent systematic reviews have evaluated the efficacy and toxicity of applying SBRT to head and neck cancers [7,8]. There is a lack of evidence supporting the use of SBRT in head and neck cancers other than in patients who are ineligible for standard treatments; however, these reviews have found that SBRT may be a promising modality in the treatment of head and neck cancers.

For OPSCCs, the experience of using SBRT is mainly limited to a boost after intensity-modulated radiotherapy, reirradiation for disease recurrence, or for post-operative patients [9-16]. While there is currently limited retrospective data describing the use of primary SBRT in OPSCC in patients who were not eligible for standard treatments, the sample sizes in these studies were small, and they included other head and neck cancers [17-20].

Additionally, the subgroup of human papillomavirus (HPV)-related OPSCC has been shown to have improved prognosis after treatment [21,22]. This has led to a recent push to de-escalate definitive treatment for this patient population by reducing the total radiation dose or modifying the chemotherapy regimen [4,23-25].

There are currently ongoing prospective randomized trials evaluating the use of SBRT for various head and neck cancer scenarios, including for elderly patients and de-escalation for HPV-related OPSCC [26-28]. Both populations may benefit from the lower anticipated toxicity profile and easier logistics of SBRT.

Here we describe a case of OPSCC that was treated with SBRT monotherapy, with subsequent pathologic confirmation of complete response.

## Case presentation

A 51-year-old female presented in 2018 with palpable lymphadenopathy in the left neck at levels II and III. Initial investigation included a CT of the neck, which showed a 2.1x1.8 cm level II lymph node and a 2.4x1.5 cm level III lymph node. Fine needle aspiration (FNA) of the cervical lymph node and panendoscopy were performed. The FNA of the left neck lymph node was consistent with squamous cell carcinoma. The left base of the tongue that was biopsied during panendoscopy showed non-keratinizing invasive squamous cell carcinoma with diffusely strong p16 positivity. PET-CT scan revealed increased uptake in the enlarged level II and III left-sided lymph nodes with a standardized uptake value of 11 and 10.4, respectively. No additional abnormalities were identified in the neck, and there was no evidence of distant metastatic disease. The patient’s staging was cT2N2bM0, stage IVA according to the American Joint Committee on Cancer (AJCC) version 7. According to the AJCC 8th edition, the staging for this HPV-related OPSCC would be cT2N1M0, stage I.

The patient’s past medical history included rheumatoid arthritis (RA) on sarilumab, claustrophobia, atrial fibrillation, resected basal cell carcinomas of the face without adjuvant therapy, previous gastrointestinal bleed secondary to corticosteroid therapy, previous deep vein thrombosis, and a current smoker with a 15-pack-year history.

Her case was reviewed in a multidisciplinary clinic, including radiation oncology, head and neck surgery, and medical oncology. Standard curative treatments were presented, including concurrent chemoradiation of 70 Gy over 35 fractions to the gross disease and elective bilateral cervical lymph node regions. She was also offered definitive intent radiation alone and surgery. However, the patient declined curative intent treatment due to fear of toxicity and was discharged with palliative care.

Four months later, palliative care referred the patient back to radiation oncology for consideration of palliative radiation for low back pain, suspected to be from bone metastases. Bone scan and staging CT scans of the head, neck, chest, and abdomen did not reveal any osseous metastasis or any other sites of metastatic disease. Instead, these scans revealed a slight increase in nodal mass sizes, with a 3.2 x 2.2 x 3.8 cm left-sided level II nodal mass and a 2.1 x 1.4 x 3.3 cm level III nodal mass. There was no new cervical lymphadenopathy or primary neoplasms. On examination, the patient had fullness in the left base of the tongue region and palpable cervical lymphadenopathy corresponding to the imaging abnormalities.

After discussing the options for treatment, the patient again declined standard curative treatment due to concerns regarding treatment toxicity. She opted for palliative SBRT to the oropharynx and unilateral left neck with a prescribed dose of 40 Gy in five fractions to the gross disease and 25 Gy in five fractions to the ipsilateral elective neck, given once per week. The radiotherapy plan was planned using the Pinnacle 3 (Phillips Medical Systems, Madison, Wisconsin) system and delivered via a Varian linear accelerator with a 6 MV volumetric-modulated arc therapy plan. A thermoplastic mask was used for patient immobilization. Radiation treatment volumes were contoured on a CT planning simulation with intravenous contrast. Due to the patient’s history of claustrophobia and palliative intent, MRI imaging was not performed.

**Figure 1:**
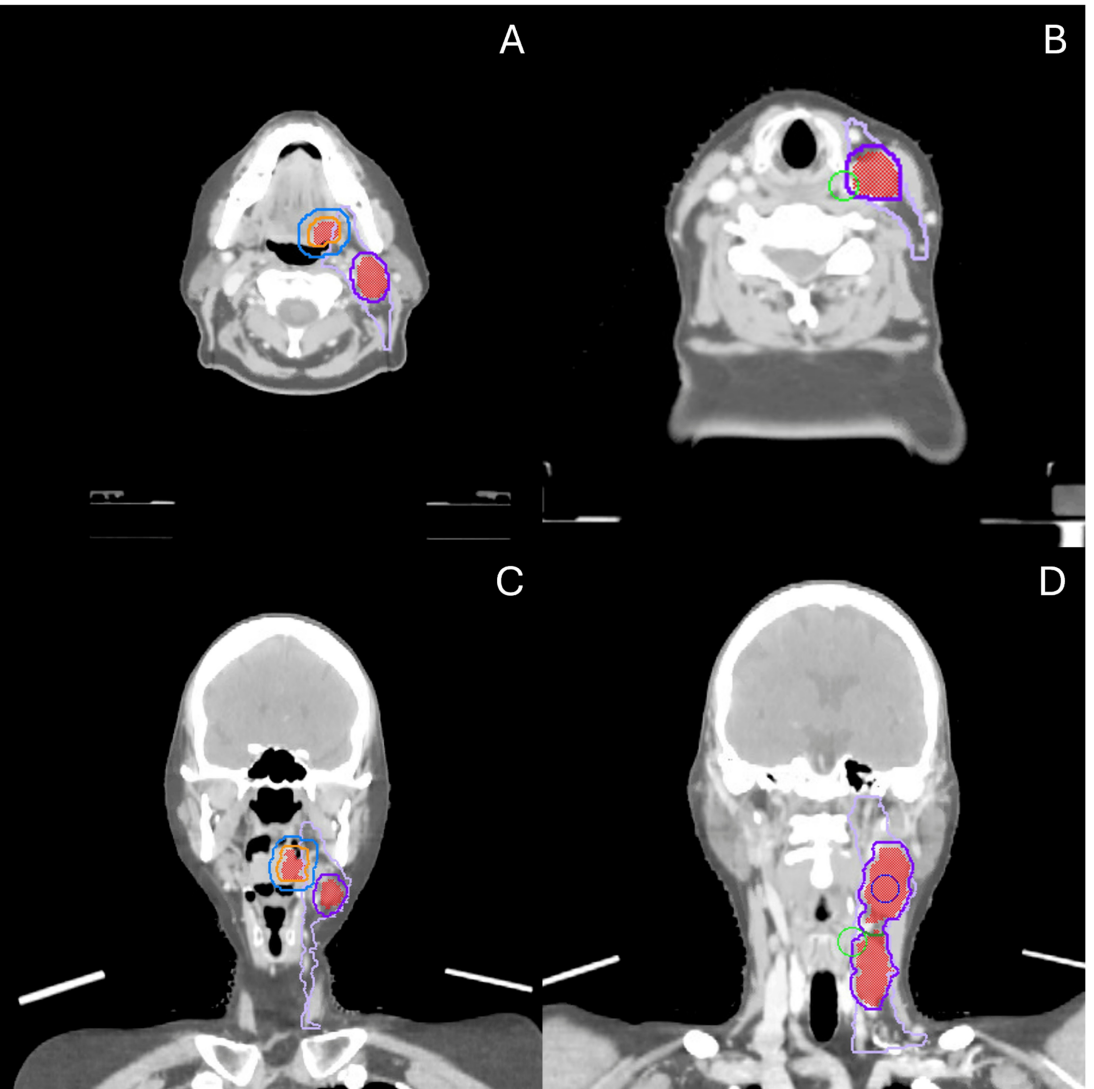
Delineated target volumes for the head and neck SBRT The red color wash denotes the primary and nodal GTV. Orange outline denotes CTVp40 and purple CTVn40, which were 2 mm expansions on the respective GTVs. Blue outline denotes CTVp25, which was a 5 mm expansion on the CTVp40, and lilac denotes the CTVn25, which was the ipsilateral elective neck levels II-IV. (A) Axial view at the level of the primary and the level II lymph node. (B) Axial view at the level of the level III lymph node. (C) Coronal view at the level of the primary and level II lymph node. (D) Coronal view at the level of the level II and III lymph nodes. CTVn25: Nodal clinical target volume prescribed 25 Gy; CTVn40: Nodal clinical target volume prescribed 40 Gy; CTVp25: Primary clinical target volume prescribed 25 Gy; CTVp40: Primary clinical target volume prescribed 40 Gy; GTV: Gross tumor volume

The gross tumor volumes (GTVs) for the left base of tongue primary (GTVp) and clinically abnormal cervical lymph nodes in left level II and level III (GTVn) were contoured by the radiation oncologist. The clinical target volumes (CTVs) for the primary (CTVp40) and nodes (CTVn40) were created by expanding the respective GTV by 2 mm in all directions, excluding uninvolved bone, muscle, skin, and air. A second CTV of the primary (CTVp25) was created by expanding the CTVp40 by 5 mm in all directions. The ipsilateral elective nodal volume, including left level II, III, and IV, was created as CTVn25 (Figure 1).

**Figure 2:**
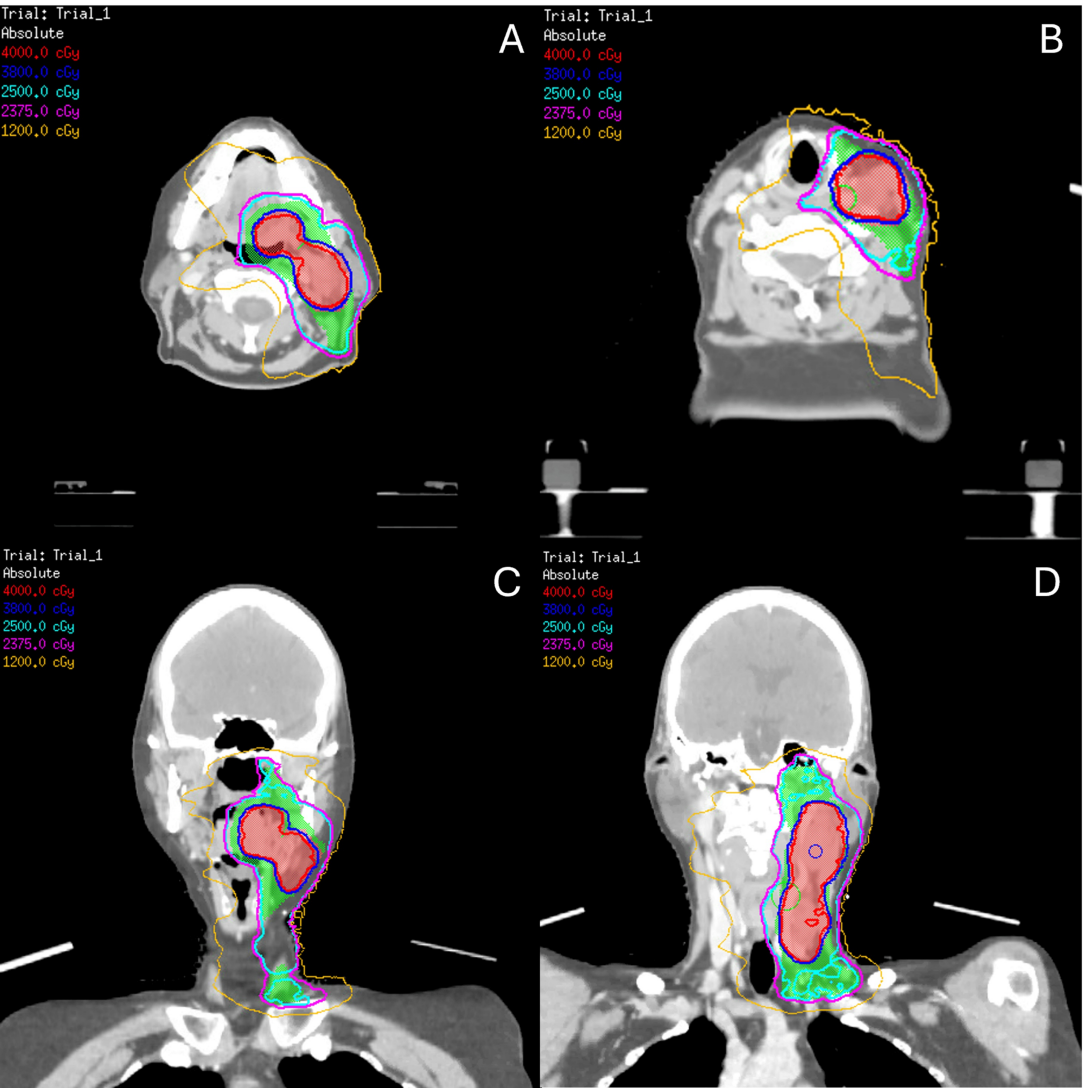
Dose distribution of the head and neck SBRT plan The red color wash represents the PTV prescribed to 40 Gy, and the green color wash represents the PTV prescribed to 25 Gy. Red isodose line represents 4000 cGy, dark blue represents 3800 cGy, teal represents 2500 cGy, fuchsia represents 2375 cGy, and orange represents 1200 cGy. (A) Axial view at the level of the primary and the level II lymph node. (B) Axial view at the level of the level III lymph node. (C) Coronal view at the level of the primary and level II lymph node. (D) Coronal view at the level of the level II and III lymph nodes. PTV: Planning target volume; SBRT: Stereotactic body radiotherapy

Planning target volumes (PTV) were made for the CTVp40, CTVp25, CTVn40, and CTVn25 by creating a 5 mm isotropic expansion on the respective CTVs. Doses of 40 Gy in five fractions and 25 Gy in five fractions were prescribed to the resulting PTVs. Figure 2 illustrates the planned dose distribution.

Dose constraints were based on the International SBRT Consortium 2016 survey for SBRT in the head and neck area [29].

She completed four out of five prescribed fractions of her radiotherapy and experienced some mild erythema of the skin, xerostomia, and moderate oral mucositis requiring short-term medicated mouthwashes and opioid analgesics. The patient opted to discontinue treatment at four fractions, at which point she had received 32 Gy to the gross tumor and 20 Gy to the elective nodal regions.

**Figure 3:**
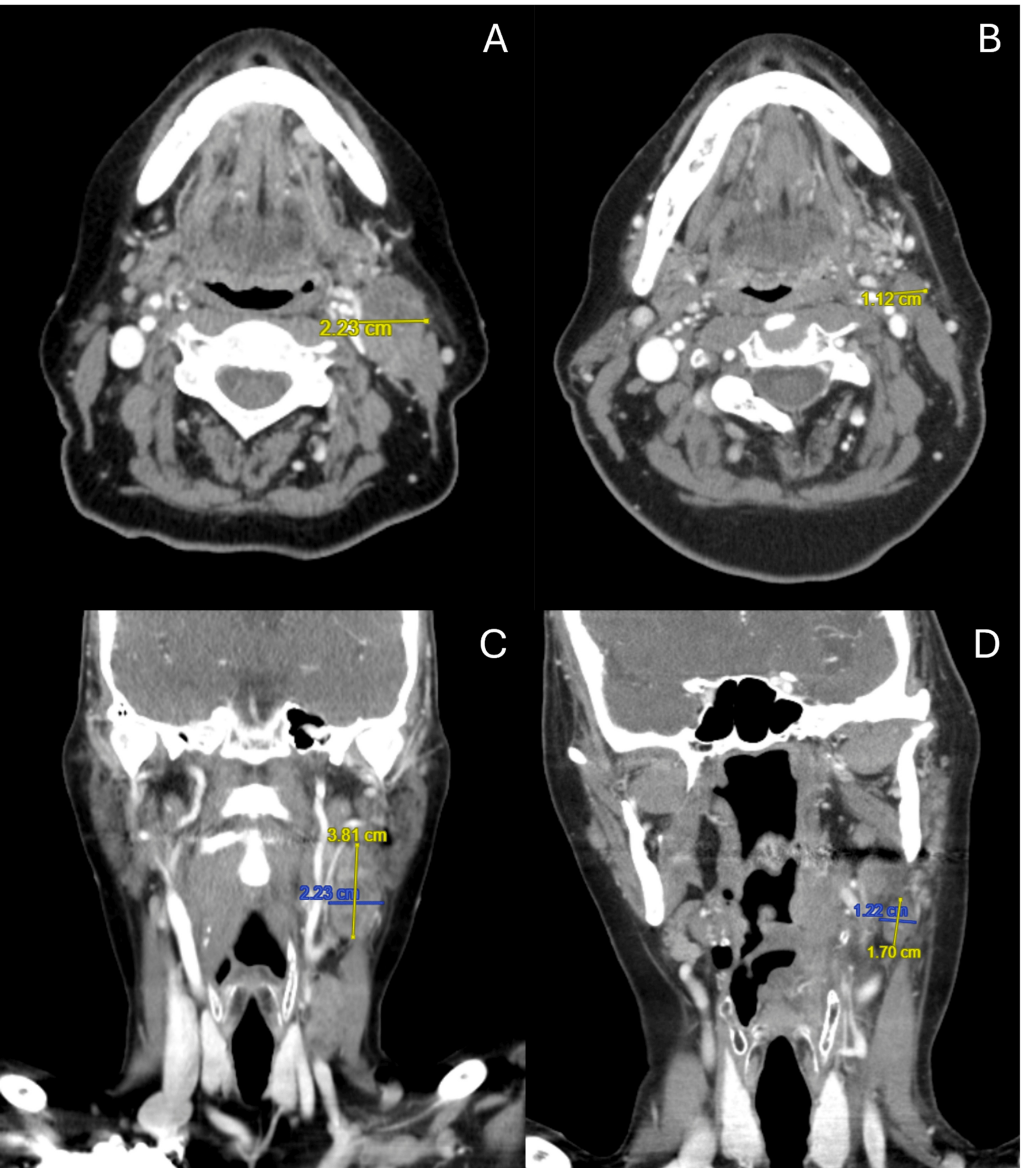
Diagnostic contrast-enhanced CT scan two months before and after SBRT Representative slices of the left level II lymph node in axial (A, B) and coronal (C, D) orientations. Panels A and C demonstrate the appearance and 2.2x3.8 cm size of the lymph node two months before SBRT. Panels B and D demonstrate the residual lymph node decreased in size to 1.2x1.7 cm two months after SBRT. There was a resolution of the treated level III lymph node in the two months after SBRT CT. The primary disease was initially CT occult. SBRT: Stereotactic body radiation therapy

At the five-week follow-up, the patient had mostly recovered from the acute side effects, with residual xerostomia and mild oral mucositis that did not require medical interventions. A follow-up CT neck and chest at approximately two months after treatment found a reduction in the level II left-sided lymph node to 1.7x1.2 cm and resolution of the level III node with no metastatic disease. There was also mild post-radiation thickening in the primary left base of the tongue and vallecula. Figure 3 depicts the left level II lymph node two months before and after SBRT.

Head and neck surgery assessed her for the residual enlarged level II lymph node and offered a PET-CT scan to assess for residual disease. The patient declined the PET scan but opted to proceed with a selective left neck dissection from levels II to IV and panendoscopy with forceps biopsy of the left base of the tongue five months after SBRT. All clinically questionable areas were sampled. Pathology showed a complete response with a necrotic and calcified level II lymph node. Fifteen other dissected lymph nodes and the sample from the left base of the tongue were benign on pathology. CT of the neck and chest performed one year after the completion of radiotherapy revealed no recurrent or metastatic disease.

At approximately 16 months after the completion of her SBRT, flexible nasopharyngoscopy showed a small 1-2 mm exophytic lesion on the left base of the tongue. This was subsequently biopsied with cupped forceps during panendoscopy and revealed focal dysplastic squamous mucosa with no invasive carcinoma. No other lesions were observed in the oral cavity, oropharynx, larynx, esophagus, or trachea.

She continued to be followed by radiation oncology and head and neck surgery. She had interval CT neck and chest up to 68 months after SBRT that continued to be negative for disease recurrence or metastasis. At 60 months after SBRT completion, she was discharged from the care of radiation oncology. Her residual post-radiation symptoms included mild fibrosis of the left neck, mild xerostomia, and non-specific dysphagia that did not affect her daily activities and did not require medical interventions. She had another flexible nasolaryngoscopy, bronchoscopy, and esophagogastroscopy performed by head and neck surgery at 68 months after SBRT for the non-specific dysphagia that did not reveal any suspicious lesions or abnormalities.

## Discussion

To our knowledge, we describe the first reported case of HPV-related oropharyngeal OPSCC that was treated with definitive SBRT and subsequent pathologic confirmation of complete response.

A literature search on PubMed to identify reports on the use of SBRT in OPSCC was performed (Appendix). Treatment volumes for the head and neck SBRT have been described heterogeneously in the literature. These vary from having the GTV as equal to the PTV with no expansion, to creating a PTV from a 2-5 mm expansion of the GTV for positional soft tissue uncertainty [12,13,30]. There is also variation in how the volumes were contoured. Vargo et al. reported using CT imaging for volume delineation similar to the case we present [19], while Al-Assaf et al. and Kodani et al. used MRI in volume delineation [18,20]. While some treatment plans reported by Al-Assaf et al. and Gogineni et al. had adjacent lymph node regions treated electively with a dose of 25-30 Gy, similar to the patient we present in this case, there were no reports of a GTV expansion to create a CTV when treating gross disease [17,18]. In our reported case, a CTV expansion was added onto the GTV for a disease that may not be well appreciated on CT imaging, as well as for microscopic disease. To maximize locoregional control, CTV expansions on the GTV similar to conventional fractionation regimens for HPV-related OPSCC were used in our patient [18,21,22,31].

In the studies that included SBRT as a primary modality of treatment for OPSCC, dose fractionation schemes varied from 30 Gy to 50 Gy in four to six fractions [17-20]. While the specific dose fractionations were not explicitly described in all studies, the EQD2 radiation prescriptions with an alpha or beta of 10 spanned from 40 Gy to over 70 Gy. There was also variation in these studies in the treatment schedule. Kodani reported patients receiving treatment daily [20], while Al-Assaf reported treatment up to twice weekly [18]. In the studies by Vargo et al. and Kodani et al., concurrent cetuximab or other systemic therapies with radiation treatment were also described [19,20].

The patient presented in this case had an initial planned radiation prescription of 40 Gy in five fractions to the gross tumor site weekly. Even though treatment was discontinued at a delivered dose of 32 Gy in four fractions, both the initial prescribed dose and delivered dose still fall within the reported dose fractionation ranges in the literature.

Table 1 demonstrates the differences in dose, volume, elective nodal coverage, use of concurrent systemic therapy, and the local control for selected head and neck SBRT studies compared to our study. Notably, studies reported local controls of 69%-82% at 12 months. 

**Table 1:** Characteristics of the selected head and neck SBRT studies compared to the present report CTV: Clinical target volume; GTV: Gross tumor volume; LC: Local control; PTV: Planning target volume

Study	Median dose to gross disease (range)	Volume	Elective lymph node coverage	Concurrent systemic therapy	Response rate
Gogineni et al. [17]	37.5 Gy (35-40 Gy/5 fractions twice weekly)	PTV = GTV + 2 mm	Yes, if gross nodal involvement	Allowed	12-month LC: 73%
Al-Assaf et al. [18]	41. 6 Gy (35-50 Gy/4-6 fractions weekly or twice weekly)	PTV = GTV + 3-5 mm	Yes, at the discretion of the treating physician	No	12-month LC: 82.1%
Vargo et al. [19]	44 Gy/5 fractions alternating days	PTV = GTV + 2-5 mm	No	Allowed	12-month LC: 69%
Current study	32 Gy/4 fractions delivered weekly (40 Gy/5 fractions planned)	CTV = GTV + 2 mm; PTV = CTV + 5 mm	Yes	No	Disease-free at 68 months

While clear phase III prospective randomized data on radiation dose de-escalation for HPV-related OPSCC are lacking, some studies suggest that HPV-related OPSCC may be adequately controlled with a total dose of less than 70 Gy given in 2 Gy per fraction. Yom et al. demonstrated a 90.5% two-year progression-free survival with concurrent chemoradiation to a dose of 60 Gy in 30 fractions [4]. Alfaraj et al. reported that in patients who did not complete planned conventional radiotherapy, the TD50 for locoregional control corresponded to a lower EQD2 dose of 23.5 Gy in HPV-positive patients compared with 62.3 Gy in patients who were HPV-negative [32]. At three years, this same study found that the overall survival rate was 94% for patients with HPV-related disease who received a BED10 ≥55 Gy, which was significantly improved from the 47% rate of overall survival in patients who received a BED10 <55 Gy. Our patient received a dose that corresponded to a BED10 of 57.6 Gy, but without sensitizing chemotherapy. In the adjuvant setting, Riaz et al. reported de-escalation of radiation dose to an EQD2 of 30 Gy with concurrent chemotherapy, which may adequately treat HPV-related OPSCC, and with subsequent pathologic complete response in a specific subset of patients without tumor hypoxia [33].

The studies that included OPSCC treated with SBRT reported a median follow-up of six to 16 months [18-20]. While the data were reported in aggregate for various head and neck cancers, complete response rates ranged from 32.5% to 38%, determined clinically or radiographically. The patient reported in this case was confirmed to have a complete pathologic response five months after the completion of her SBRT, with no signs or symptoms or disease recurrence at 68 months after SBRT.

The Common Terminology Criteria for Adverse Events v5.0 grading scale was used to determine retrospective toxicity grading. The patient presented in this case was known to have RA on biologic medication. A recent systematic review by Liebenberg et al. describes the impact of RA on radiation toxicity. They describe that patients who have RA may have a higher rate of late toxicity but without enough high-quality evidence to warrant radiation dose reductions [34]. The patient in this case presented grade two dry skin, grade two oral mucositis, and grade one salivary duct inflammation acutely. Late radiation side effects included grade one salivary duct inflammation and grade one superficial and deep connective tissue fibrosis. There were no grade three or greater toxicities, in line with previous reports of improved tolerability and reduced toxicity of the SBRT treatments [18-20].

While this patient had a pathologic complete response to SBRT monotherapy, the EQD2 for the tumor was on the lower end of the previously published EQD2 values for the tumor. It is important to note that in the above-discussed studies investigating the use of SBRT, the HPV status and p16 positivity of OPSCC were not reported.

A limitation of this report includes the type of biopsy performed on the primary base of tongue lesion. While a neck dissection clearly demonstrated no pathologically viable tumor in the ipsilateral neck after SBRT, the base of the tongue was only sampled via forceps biopsy. This was performed to avoid the significant morbidity of glossectomy, but it does open the possibility of sampling error. We believe that a false-negative result is unlikely, given the two sets of negative biopsies at five and 16 months after SBRT, and long-term clinical and radiographic response at 68 months after SBRT.

Several ongoing prospective trials are evaluating SBRT for head and neck cancers. Some of these include the SHINE study evaluating the safety and efficacy for elderly patients with head and neck cutaneous or mucosal SCC patients, the SHORT-OPC trial investigating the local control of de-intensified SBRT boost compared with conventional radiation for p16-associated OPSCC, and HN13 evaluating overall survival of SBRT for mucosal head and neck SCC patients unable to tolerate curative intent treatment [26-28]. The results of these trials are highly anticipated to better understand the appropriate patients and the safety and efficacy of SBRT for head and neck cancers.

## Conclusions

In this report, we describe a case of locally advanced HPV-related OPSCC treated with SBRT, resulting in a complete pathologic response and no evidence of recurrent cancer at over five years of follow-up. Our technique included treatment of the gross primary and nodal disease volumes with 2 mm CTV expansions to full dose, in addition to treating the ipsilateral neck with elective nodal radiation. There is significant interest in both the de-escalation of definitive radiation treatment for HPV-related OPSCC and the use of SBRT in head and neck malignancies for patients who may not tolerate definitive intent chemoradiation. The case presented in this report provides some evidence that SBRT monotherapy can provide durable locoregional control and even pathologic complete response for a patient who was fit for but declined standard therapy. This case was, however, highly selected and should not be generalized to other patient populations until proven by clinical trials. Ongoing points of interest with regard to head and neck SBRT include dose, fractionation, treatment volumes, appropriate patient selection, toxicity, and efficacy. Ongoing and future clinical trials are warranted to provide level one evidence supporting SBRT for head and neck cancers.

## Appendices

PubMed literature search terms and dates

Aug 18, 2024: ((((((body radiotherapies, stereotactic) OR (body radiotherapy, stereotactic)) OR (radiosurgeries, stereotactic)) OR (radiosurgery, stereotactic)) OR (radiotherapies, stereotactic body))) AND (oropharyngeal neoplasms)

Mar 11, 2021: “oropharyngeal stereotactic radiation”, ("oropharyngeal neoplasms"[MeSH Terms]) AND (((((body radiotherapies, stereotactic[MeSH Terms]) OR (body radiotherapy, stereotactic[MeSH Terms]) OR (radiosurgeries, stereotactic[MeSH Terms]) OR (radiosurgery, stereotactic[MeSH Terms]) OR (radiotherapies, stereotactic body[MeSH Terms])))))
